# Pulsed 3.5 GHz high power microwaves irradiation on physiological solution and their biological evaluation on human cell lines

**DOI:** 10.1038/s41598-021-88078-x

**Published:** 2021-04-19

**Authors:** Pradeep Bhartiya, Sohail Mumtaz, Jun Sup Lim, Neha Kaushik, Pradeep Lamichhane, Linh Nhat Nguyen, Jung Hyun Jang, Sang Ho Yoon, Jin Joo Choi, Nagendra Kumar Kaushik, Eun Ha Choi

**Affiliations:** 1 Plasma Bioscience Research Center/Applied Plasma Medicine Center, Department of Plasma Bio Display, Department of Electrical and Biological Physics, Kwangwoon University, Seoul, 01897 Korea; 2 College of Engineering, Department of Biotechnology, University of Suwon, Hwaseong, 18323 Korea

**Keywords:** Cell biology, Physics

## Abstract

Microwave (MW) radiation is increasingly being used for several biological applications. Many investigations have focused on understanding the potential influences of pulsed MW irradiation on biological solutions. The current study aimed to investigate the effects of 3.5 GHz pulsed MW radiation-irradiated liquid solutions on the survival of human cancer and normal cells. Different physiological solutions such as phosphate buffer saline, deionized water, and Dulbecco’s modified Eagle medium (DMEM) for cell culture growth were irradiated with pulsed MW radiation (45 shots with the energy of 1 mJ/shot). We then evaluated physiological effects such as cell viability, metabolic activity, mitochondrial membrane potential, cell cycle, and cell death in cells treated with MW-irradiated biological solutions. As MW irradiation with power density ~ 12 kW/cm^2^ mainly induces reactive nitrogen oxygen species in deionized water, it altered the cell cycle, membrane potential, and cell death rates in U373MG cells due to its high electric field ~ 11 kV/cm in water. Interestingly, MW-irradiated cell culture medium and phosphate-buffered saline did not alter the cellular viability and metabolic energy of cancer and normal cells without affecting the expression of genes responsible for cell death. Taken together, MW-irradiated water can alter cellular physiology noticeably, whereas irradiated media and buffered saline solutions induce negligible or irrelevant changes that do not affect cellular health.

## Introduction

Microwaves (MW) have been extensively used in daily life depending on their specific frequency, power, and properties. MW can propagate through substances, air, and vacuum. These radiations can be classified into two types: ionizing and non-ionizing radiations. Non-ionizing radiation lacks the energy required to break the atomic or chemical bonding of molecules in biological cells. MW radiation and heating has been used for domestic, industrial (agriculture, telecommunications, advanced military equipment), and medical purposes^1,2^. MW heating differs from conventional heating because it involves dipolar polarization, rotation, and alignment of molecules with respect to the applied microwave field. In addition, MW heating is selective, non-uniform, and largely dependent on the properties of the material. Materials in solution or having a polar nature absorb MW efficiently and transform it into heat. This heat accounts for the hyperthermia-related effects of MW. Direct and indirect exposure to MW radiation induces hyperthermia and can facilitate many biologically beneficial and detrimental effects^3,4^. Methods involving indirect exposure or irradiation with MW are largely employed in the food industry and in the extraction of compounds^5–8^. MW can be used widely in biological systems for different purposes including staining, biological tissue processing, biomedical imaging, extraction, disinfection and sterilization, waste treatment, cancer detection, protein folding and unfolding, protein hydrolysis and proteomics, methane production, moisture removal, enzyme immobilization, and mutagenesis in plants^2,9–12^. MW imaging and sensing have been used in the detection of early tumor stages, blood clot/stroke detection, early diagnostics, bone imaging, and heart imaging^13–20^. MW have also been used for detecting early stage breast cancer using the dual-mode or thermoacoustic imaging model. Continuous exposure to MW could be useful for biological functions such as increased wound healing and intracranial hematoma detection^13–20^. Both thermal and non-thermal MW have been explored for their effects on cellular and even single protein activity levels^21,22^. On the contrary, MW also have extensive health-damaging effects, mostly on the human nervous system^23–28^. Pulsed and dose-dependent MW affects immunomodulation and autoimmunity by inducing morphological and functional injuries in natural killer (NK-92) cells, probably through perforin expression and ERK-mediated apoptosis regulation^29^. Continuous exposure to a 900-MHz electromagnetic field for 1 h (h) in a day has been shown to impair the normal morphological cell structures, with changes in biochemical parameters, and elevation of apoptosis^30^. Pulsed MW has been investigated for its effects on bone marrow cells at a frequency of 2.856 GHz, but showed no significant changes in cell viability, apoptosis, and cell division^31^. Overall, considering the increasing applications of MW in biological and other aspects of routine life, it is imperative to understand and determine the effects of MW on biological systems^1^.

Among many applications, MW heating of sample materials in solutions or solvents results in many unique changes (referred to as microwave specific effects) and solvent overheating, which are not achieved by conventional heating methods. MW can exert such unique non-thermal effects by storage of energy either as vibrational energy of molecules or functional groups, or by molecular alignment resulting from the selective heating of materials in a biological/chemical solution^32–35^. While several reports support this phenomenon of MW by demonstrating induction of cell death due to electric fields or low temperatures^36,37^, other studies present opposing views on the effects of non-thermal specific effects^38,39^. This has thus become a controversial topic and needs further investigation for better understanding of the effect of MW on solutions or biological systems. MW irradiation of solutions can alter solution properties temporarily by storing energy within solutions and can induce significant changes in protein function and thus cellular function^40,41^.

Thus, it is imperative to study MW radiation and interaction mechanisms in solutions for beneficial applications in biological systems. In this study, we aimed to observe the effect of pulsed high-power microwave (HPM)-irradiated solutions on human-derived normal and cancer cells. These effects were assessed at the cellular level by measuring metabolic viability and cell death. MW radiation can vary significantly based on the composition of the solution^42,43^. In this context, cell culture media, deionized water (DW), and phosphate buffered saline (PBS) solutions were used throughout all the experiments. Media are used for normal cell growth in biological systems and consist of macromolecules such as proteins and lipids, whereas PBS and DW are the most common physiological solutions used in biological experiments.

## Results

### Physical characteristics of the HPM exposure device

**Figure 1:**
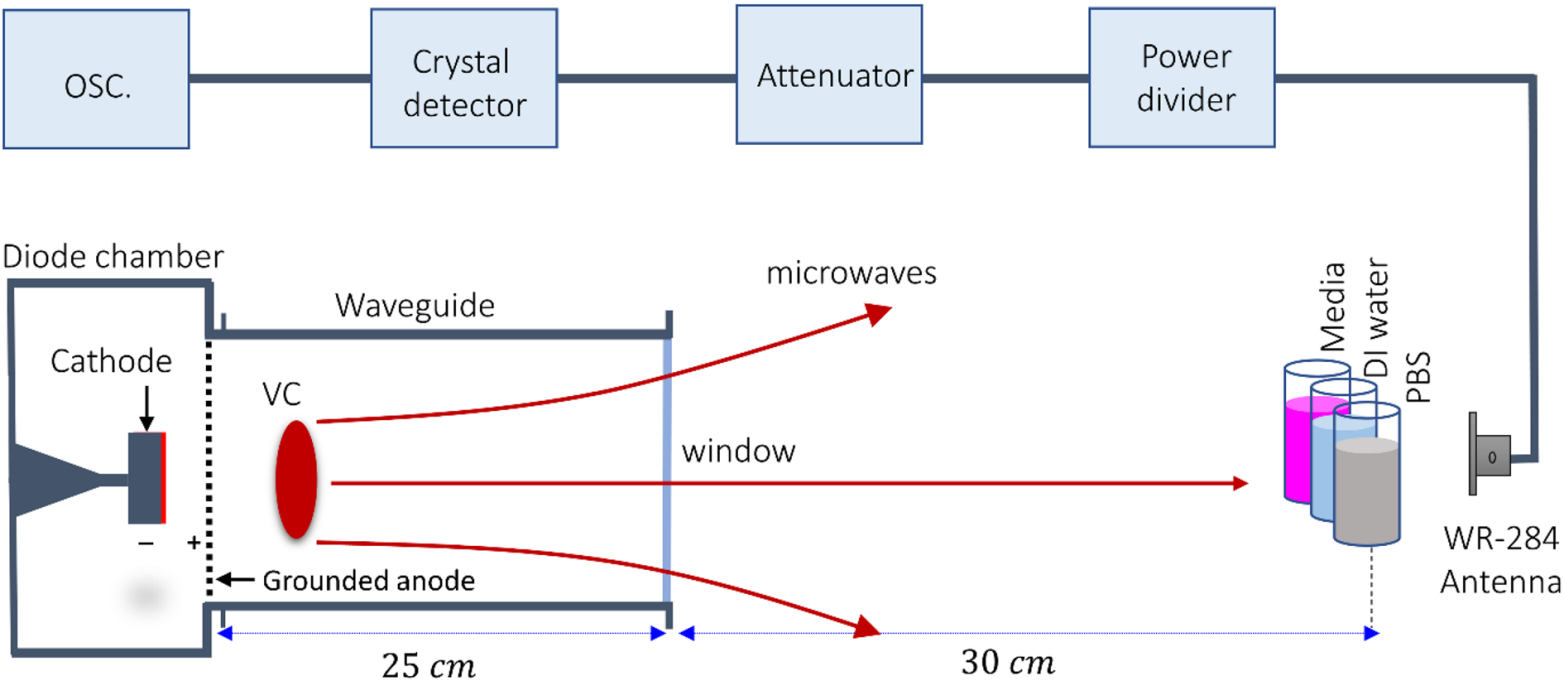
Configuration of the diode region of the HPM generator “Chundoong”. The emitter cathode used for the emission of electron beam. The anode and cathode gap distance was 10 mm and the high power microwaves (HPM) were generated with a frequency of 3.5 GHz from the dense virtual cathode (VC) and propagated outside from the window of the waveguide. The bilological solutions were positioned 30 cm away from the window. The WR-284 is the receiving antenna which is used to measure the power of HPM. The HPM then passes through the power divder to divde the power into two equal amounts, attenuator, and a crystal detector by coaxial wire for the traces of the waveforms at oscilloscope in the electromagnetically shielded room**.**

An axial vircator was constructed to generate HPM from the virtual cathode (VC). Figure 1 shows the configuration of the vacuum diode region of the HPM exposure device “Chundoong” used in this work. Briefly, Chundoong consists of a cathode made of aluminum with a radius of 4.5 cm covered with a velvet layer, and an anode made of stainless-steel mesh with a radius of 10 cm. The waveguide was 25 cm long, and its inner radius was 10 cm. The diode chamber and DT regions were evacuated with vacuum pressure up to 1 × 10^−5^ Torr in this experiment. The end part of the drift tube was sealed by an acrylic window with a thickness of 1.5 cm to maintain the vacuum pressure in the diode. This window also allowed the generated HPM to radiate and avoid surface breakdown outside the DT. The samples (media, DW water, and PBS) were placed 30 cm away from the window where they were irradiated with HPM using 45 shots of the electromagnetic energy “$${\text{E}}$$”, which is calculated as $${\text{E}} = {\text{P }} \times {\raise0.7ex\hbox{${\text{t}}$} \!\mathord{\left/ {\vphantom {{\text{t}} 2}}\right.\kern-\nulldelimiterspace} \!\lower0.7ex\hbox{$2$}} \cong 1{\text{ mJ}}$$ per trigger shot, where $${\text{P}}$$ is the HPM power reaching the sample, and $${\text{t}}$$ ~ 60 ns is the pulse duration. Hence, 1 mJ electromagnetic energy was delivered to the sample at each trigger shot every 1 min.

**Figure 2:**
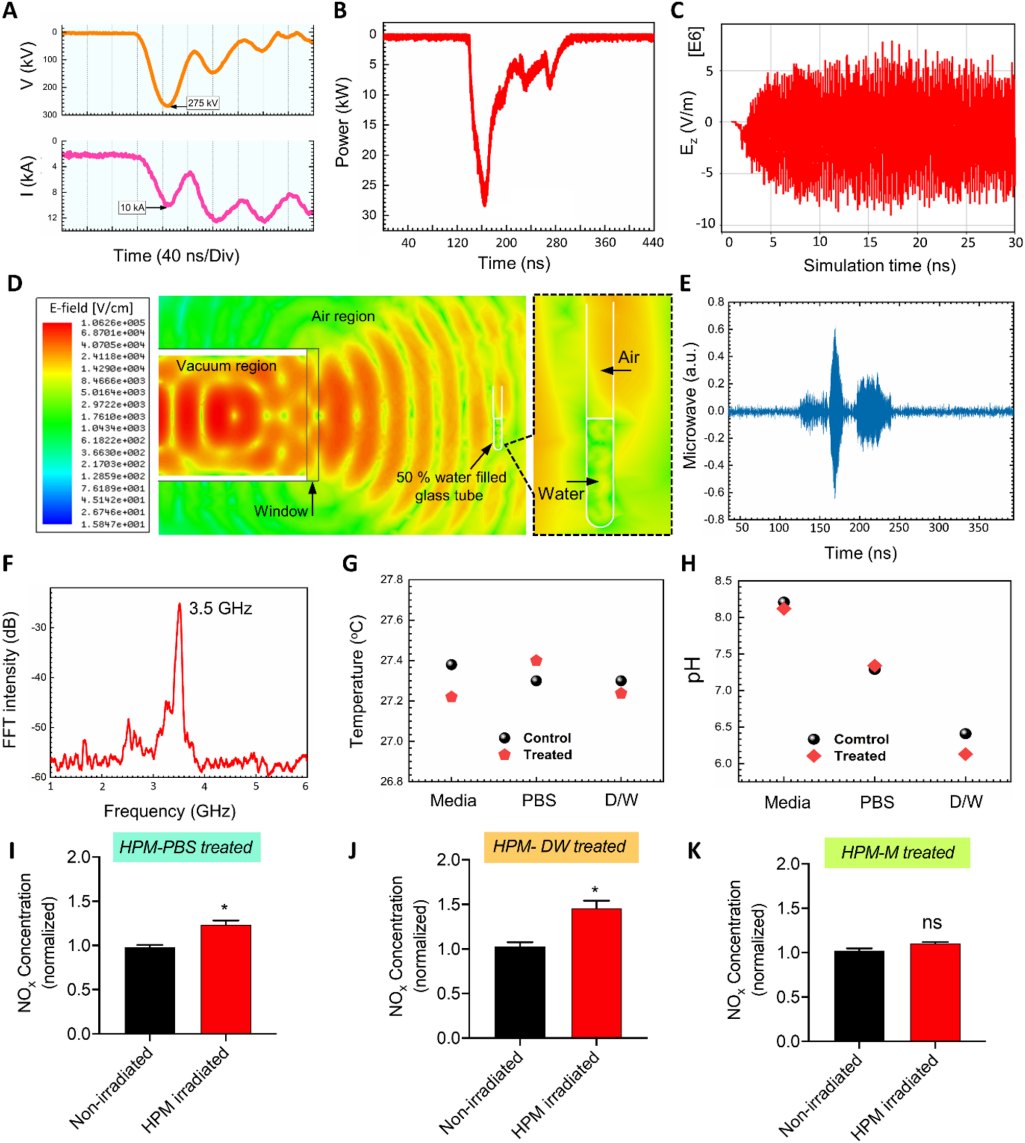
Physical characteristics of the high power microwave exposure device “Chundoong”. (**A**) Diode voltage and diode current, (**B**) Microwave envelope signal, (**C**) Axial electric field from simulation, (**D**) Electric field from HFSS simulation, (**E**) Microwave realsignal, (**F**) Dominant frequency of the microwaves, (**G**) Temperature of the solution before and after microwave exposure, (**H**) pH of the solution before and after microwave exposure, and NO_x_ concentration in (**I**) PBS, (**J**) water, and (**K**) cell culture media.

The physical characteristics of the HPM were measured and analyzed. Figure 2A show the diode voltage and diode current with peak values of 275 kV and 10 kA, respectively. The sample was positioned 30 cm away from the window. At each trigger shot, an HPM pulse was generated from the VC and radiated in the atmosphere from the output window, and propagated toward sample^44^. The mechanism for generating HPM from the virtual cathode has been described in previous studies^44,45^. The sample was exposed to HPM with an average power of 30 kW^46^, as shown in Fig. 2B, of the HPM envelope signal, which corresponds to ~ 1 mJ electromagnetic energy delivered to the liquid sample at each trigger shot. **T**he HPM power 30 kW is measured to be almost constant along with axial distance from the exit of the window (z = 0) to the position of the sample (z = 30 cm). Here, the electric field strength, E_max_, has been estimated to be ~ 11 kV/cm from the HPM energy flowing Poynting vector, S, reached to the interaction area A = 2.5 cm^2^ at the sample, from which the time averaged HPM power, P_avg_, could be expressed by P_avg_ = SA, where P_avg_ = 30 kW, S = E_max_^2^/2*u*_*o*_c is Poynting power density, *u*_*o*_ = 4π × 10^–7^ T·m/s is magnetic permeability in vacuum, and c = 3.0 × 10^8^ m/s is speed of light. The Poynting power density, S, is estimated to be ~ 12 kW/cm^2^ in this experiment. Also, the electric field with a strength of ~ 11 kV/cm, which is found to be similar to that conventional nonthermal atmospheric pressure plasma jet^47^, interacts with the air–liquid solution interfacial region at each HPM pulse**.** Figure 2C shows the axial electric field $$E_{z}$$ inside the drift tube region from the particle in cell simulation code, “MAGIC”. The maximum magnitude of $$E_{z} $$ is found to be ~ 70 kV/cm inside the drift tube. By taking into account far field pattern of electric field in HPM, the magnitude of electric field would be decreased to ~ 11 kV/cm at the sample position (z = 30 cm) from ~ 70 kV/cm in the vacuum drift tube region, by the $$1/r $$ dependence of far-field pattern and power loss due to the spreading of HPM (here, $$r$$ is the distance from acrylic exit window of HPM to the target sample). The electric field distributions at the region of z = 30 cm with biological sample could be obtained by HFSS (High-frequency structure simulator) code, as shown in Fig. 2D, from which both electric fields in air and water regions in test tube are found to be similar magnitude of E = 10 kV/cm to each other in this simulation study. Also the electric field distribution inside the water has shown to be in resonant nodal patterns along the test tube’s vertical direction with regularly spaced ~ 2 cm, which is exactly equal to λ/4. Here the dielectric constant *K*_*w*_ of water has been set to be around *K*_*w*_ = 77^48,49^ for electric field distributions, and magnetic permeability μ to be set to be μ = μ_o_ of air under microwave frequency of 3.5 GHz in this work. Also the boundary condition across the air and water interfacial surface, where the glass thickness is assumed to be very thin, for the radial electric field distribution is E_r,a_ = E_r,w_, which gives E_r,w_ ~ 10 kV/cm, where E_r,a_ and E_r,w_ are the radial electric fields in air and water, respectively. Also the boundary condition for the propagating component, E_z_, of electric field_,_ across the air and water interfacial surface is ε_o_E_z,a_ = 77ε_o_E_z,w_, which gives E_z,w_ ~ E_z,a_/77 ~ 130 V/cm to be negligible, where E_z,a_ and E_z,w_ are the propagating electric fields in air and water, respectively. Hence the electric field in the water, E_w_, from the HFSS, estimated to be $$E_{w} = \sqrt {E_{r,w}^{2} + E_{z,w}^{2} }$$ ~ $$E_{r,w}$$ ~ 10 kV/cm in this work, which is mainly attributed by its radial component. Here the measured electric field ~ 11 kV/cm in an air region, z = 30 cm, is similar to ~ 10 kV/cm obtained from from HFSS code in air at z = 30 cm, as well as interior of water at the same position. Hence the water and nitrogen molecular excitation would be mostly occured by HPM electric field ~ 11 kV/cm in both regions of air and interior of water in the test tube. Figure 2E shows the actual microwave signal from which the frequency of the HPM was determined to be 3.5 GHz using the fast Fourier transformation as shown in Fig. 2F. Figure 2G shows the temperature of the sample solutions (Media, PBS, DW) which is monitored by using a thermal imager FLUKE Ti9 to confirm the non-thermal effect of pulsed HPM. In this work, the temperature was monitored by placing the sample solution in the treatment room when the room temperature is 27 °C. After 45-min, the temperature of the solution was monitored in two conditions, without HPM exposure (control) and with HPM exposure (treated). The temperature of the solutions remained the same after exposure, which indicates the subsequent effects as non-thermal effect of the pulsed HPM generated from VC. The profile of the vector magnetic field, measured at the end of the drift tube during the simulation using the particle in cell simulation code, “MAGIC” shows the dominant mode of the generated HPM is TM_01_ (here, subscript 0 represents no radial node and 1 denotes polar angle dependence of cosθ) mode, which is in good agreement with previous results^44,50^. Figure 2H shows the pH of the solutions; no significant pH changes were observed after HPM exposure when compared with the control. Further, we irradiated the solutions and investigated the levels of reactive species of biological significance, such as hydrogen peroxide and nitrogen oxygen species (NO_x_). As shown in Fig. 2I and S1(supplementary), J, and K, NO_x_ levels were elevated in HPM-DW and were moderately increased in the HPM-PBS groups, whereas no such change was observed in the HPM-Media groups. Hydrogen peroxide levels were slightly increased in HPM-DW, whereas such an increase was not observed in other treatment groups (Fig S1).

### Preliminary assessment for cytotoxicity in human cells

**Figure 3:**
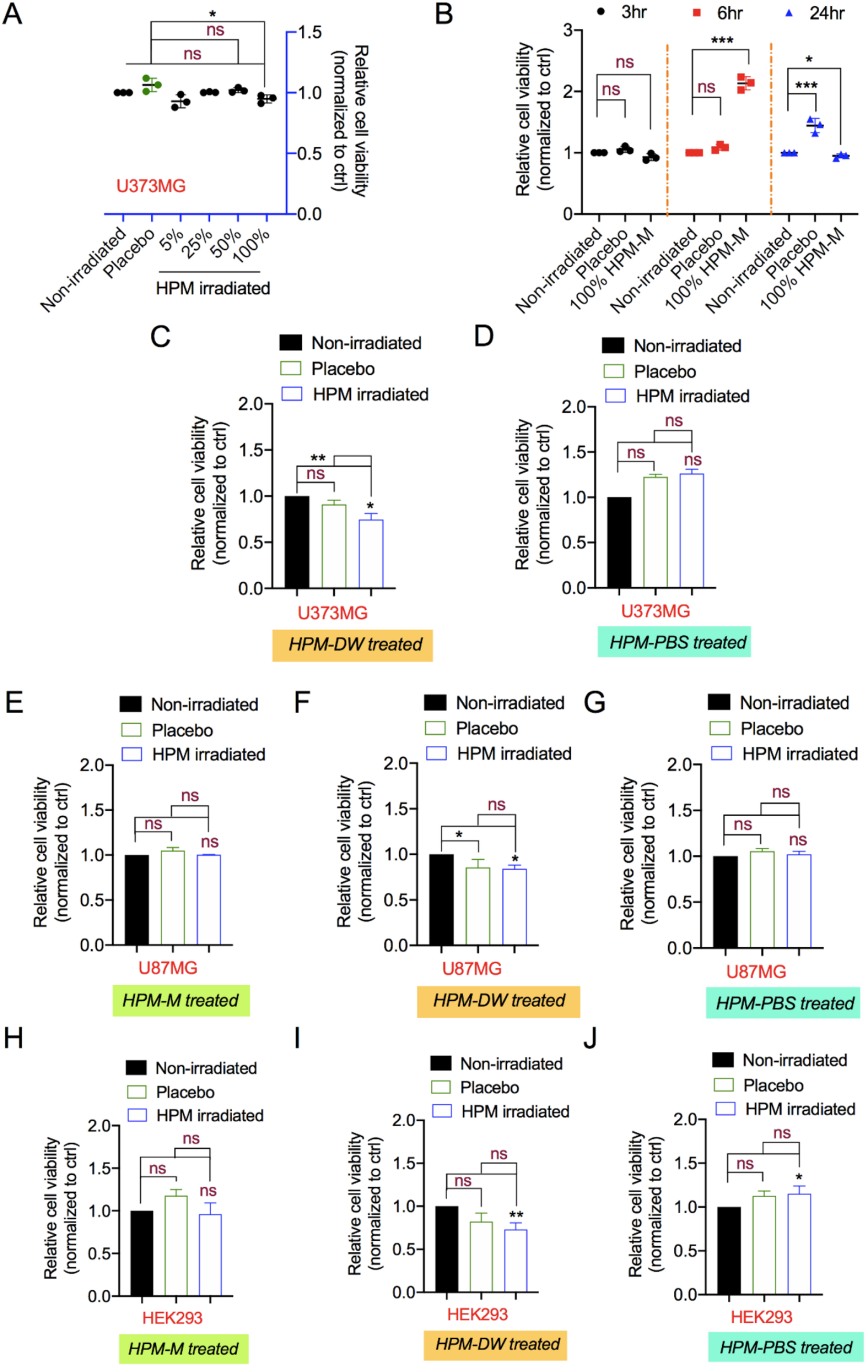
Assessment of high power microwave-irradiated solutions for cytotoxicity. (**A**) Relative viability of cells treated with increasing concentrations (5%, 25%,50%, and 100%) of high power microwave (HPM)-irradiated complete DMEM. (**B**) Relative viability of cells incubated for different times (3, 6, and 24 h) after treatment with HPM-irradiated complete DMEM (100%). Relative cellular viability measured using the Alamar Blue assay in U373MG cells treated with HPM-irradiated (**C**) Media, (**D**) DW, and (**E**) PBS. Similarly, relative viability was measured in U87MG cells treated with HPM-irradiated (**F**) Media, (**G**) DW, and (**H**) PBS, as well as in HEK293 cells treated with HPM-irradiated (**I**) Media, (**J**) DW, and (**K**) PBS. Non-significance is denoted as ns. *p < 0.05, **p < 0.01, ***p < 0.001.

Cytotoxicity and safety tests are the basic and foremost evaluations for biocompatibility and medical applications. A preliminary analysis of the effect of HPM-irradiated solutions on cells was performed by assessing their metabolic viability post treatment. For this, we tested the cancer cell line (U373MG) with increasing dilutions of HPM-irradiated complete Dulbecco’s modified Eagle medium (DMEM) (HPM-M) after 24 h incubation. The HPM-M was diluted 5–100% with normal media and added to the cells in culture. Higher dilutions (5–50%) of HPM-M did not affect the viability of U373MG cells compared to untreated media. The lowest dilution (100%) showed significant inhibition of cell viability upto 10–12% after 24 h incubation as shown in Fig. 3A. As 100% of HPM-M showed little effect on U373MG cell viability, the data indicated that the reduction in cell viability was consistently non-significant as compared to the control medium at 3, 6, and 24 h incubation (Fig. 3B). Further we investigated if treatment with HPM-DW leads to any changes in intracellular nitric oxide levels. As shown in Fig S2, U373MG cells treated with HPM-DW shows significant elevation in intracellular levels.

Taking this into consideration, we performed further experiments on U87MG cancer and HEK293 normal cell lines using deionized water (HPM-DW) and phosphate buffered saline (HPM-PBS) along with medium (HPM-M). Compared to DMEM, DW and PBS lack growth nutrients. We thus used 50% dilution of media with DW and PBS to avoid excessive hypotonic stress effects on cells^51^. In addition, placebo groups were used to assess the effects of PBS and DW without HPM irradiation. Compared to the untreated control groups, the viability of U373MG cells was significantly reduced in the HPM-DW groups (Fig. 3C), whereas it was unchanged in the HPM-PBS groups, as shown in Fig. 3D. In addition, there was a significant difference between the HPM-DW and placebo groups (Fig. 3C). For U87MG cells, groups treated with HPM-M and HPM-PBS showed no reduction in viability, as shown in Fig. 3E and G, respectively. As shown in Fig. 3F, the HPM-DW groups in U87MG cells showed a significant difference in viability reduction compared to the untreated control. However, this difference was not significant in comparison to the placebo groups. Figure 3H and J show that HPM-M and HPM-PBS treatment did not reduce the viability of HEK293 cells, whereas HPM-DW-treated groups showed a significant reduction in viability when compared with the untreated control (Fig. 3I). The effect seen in the HPM-DW groups was also observed in the placebo groups, indicating no additional effect of HPM irradiation. Taken together, these findings suggest that treatment with HPM-DW could reduce U373MG cellular viability, whereas HPM-M and HPM-PBS had little effect in these cells.

### Influence of HPM-irradiated solutions on the energetics of human cells

**Figure 4:**
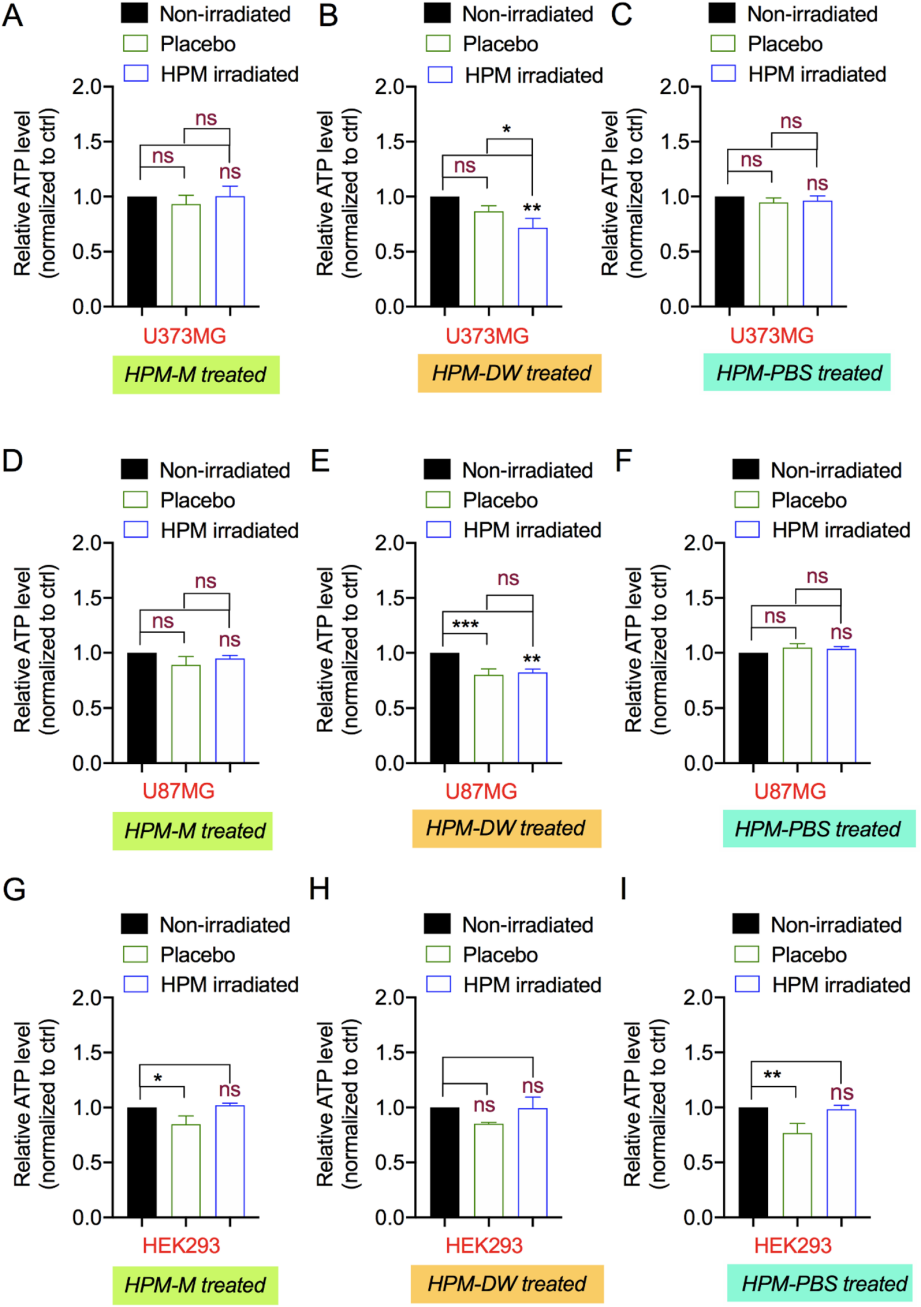
Effects of high power microwave-irradiated solutions on cellular energetics. The Cell-Titer Glo assay was used to measure ATP levels in U373MG cells treated with HPM-irradiated (**A**) Media, (**B**) DW, and (**C**) PBS; in U87MG cells reated with HPM-irradiated (**D**) Media, (**E**) DW, and (**F**) PBS; and in HEK293 cells treated with HPM-irradiated (**G**) Media, (**H**) DW, and (**I**) PBS. Non-significance is denoted as ns. *p < 0.05, **p < 0.01, ***p < 0.001.

The primary molecular effect on cells is associated with energy production and regulation. Cells maintain their energy needs by regulating adenosine triphosphate synthesis and consumption. ATP is a key regulator of signaling required for survival and proliferation. Thus, its levels can be measured as an indicator of cellular health. Therefore, we treated all three cell lines with HPM-M, HPM-DW, and HPM-PBS and measured their ATP levels. As shown in Fig. 4A and C, the ATP levels of U373MG cells remained unaffected in the HPM-M and HPM-PBS groups compared to the untreated control or placebo groups. However, the HPM-DW-treated groups showed a significant reduction in ATP levels compared to the untreated control (Fig. 4B). This effect of HPM-DW treatment on ATP reduction was also significant when compared to the placebo groups. Similar to U373MG cells, U87MG cells showed no effects with HPM-M and HPM-PBS treatments (Fig. 4D and F). However, the HPM-DW groups showed a significant reduction in ATP levels compared to the untreated control. However, this effect was similar in both the HPM-irradiated and placebo groups, indicating no significant effects of the treatment (Fig. 4E). In addition to U373MG and U87MG, normal HEK293 cells also showed no changes in ATP levels with all three HPM-M, HPM-DW, and HPM-PBS treatments compared to the untreated control, as shown in Fig. 4G–I. However, we observed slight reduction in ATP levels in HEK293 cells after treatment with placebo group only. These changes could be due to transient stress induced after treatment with placebo groups solutions (due to dilution of growth media) in these cells. Overall, HPM-DW treatment attenuated ATP levels, thus indicating cytotoxicity, in U373MG cells compared to those in the untreated control and placebo groups.

### Analysis of cell death induction by HPM-irradiated medium

**Figure 5:**
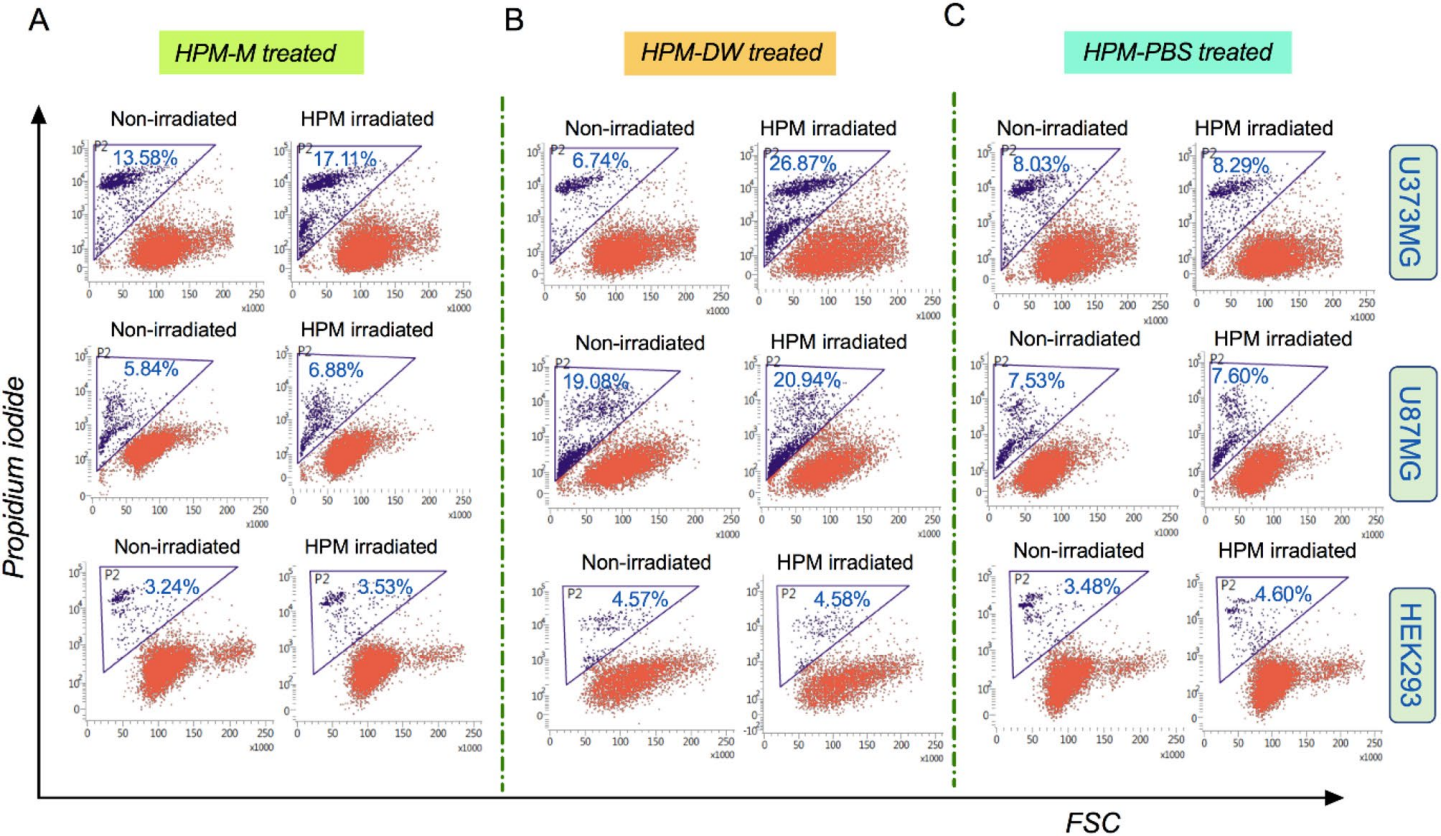
High power microwave-irradiated solutions and their impact on cell death. Representative FACS scatter plots showing cell populations bearing propidium iodide fluorescence, indicating its uptake. The groups represented are HPM-irradiated (**A**) Media, (**B**) DW, and (**C**) PBS. The data for U373MG, U87MG, and HEK293 cells are represented in the upper, middle and lower panel, respectively.

Changes in cell viability indicate cellular health and tendency towards cell survival or cell death. Reduction in ATP levels is associated with irreversible damage to the mitochondria and other cell organelles, which may lead to cell death. To determine whether treatment with HPM-irradiated solutions induced cell death, we assessed the effect of these treatments by measuring the uptake of propidium iodide (PI) in all three cell lines. Cells were harvested, stained with propidium iodide, and analyzed by flow cytometry. The increase in cell death in the HPM-M groups was found to be 3.31%, 1.04%, and 0% compared to the untreated control in U373MG, U87MG, and HEK293 cells, respectively (Fig. 5A). This difference was not significant between the HPM-M-treated and untreated groups. In U373MG cells, the HPM-DW-treated groups showed a significant increase in cell death (more than 19%) compared to that in the untreated control group (Fig. 5B-upper side). In U87MG cells, the HPM-DW groups showed no significant differences when compared to the untreated groups (1.86%) (Fig. 5B-middle side). In HEK293 cells, the HPM-DW groups showed no significant differences when compared to the untreated groups (Fig. 5B-lower side). Further, the HPM-PBS groups showed no significant cell death as compared to the untreated groups in all three cell lines (Fig. 5C).

**Figure 6:**
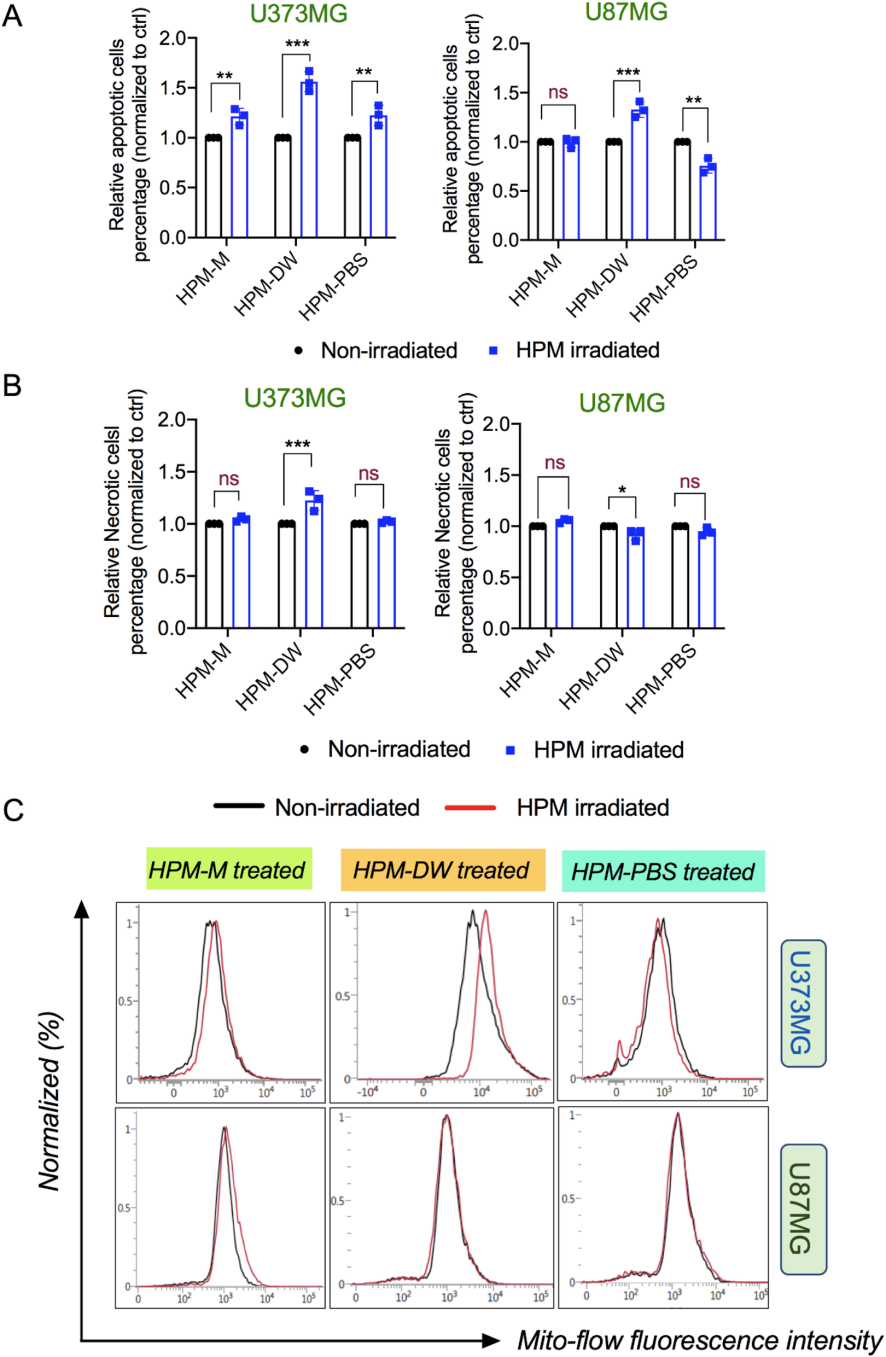
Effect of high power microwave-irradiated solutions on the mitochondrial membrane and apoptosis/necrosis. (**A**) Representative graph showing the results of an apoptosis assay in U373MG (Left) and U87MG (Right) cells with high power microwave (HPM)-irradiated media, DW, and PBS as the treatment groups (left to right respectively) (**B**) A graph indicating necrotic cell populations in U373MG (Left) and U87MG (Right) cells with the indicated treatment groups. (**C**) Representative FACS histogram plots showing the Mitoflow reagent fluorescence in U373MG (upper side) and U87MG cells (lower side) in groups treated with HPM-irradiated Media, DW, and PBS, respectively (from left to right). Non-significance is signified as ns. *p < 0.05, **p < 0.01, ***p < 0.001.

To further analyze cell death and cellular damage, we evaluated U373MG and U87MG cells for apoptosis/necrosis after treatment with HPM-irradiated solutions using the Promega Realtime-Glo Annexin V Apoptosis And Necrosis Assay kit. Cells were treated with HPM-irradiated solutions and processed according to the manufacturer’s protocol for measuring apoptosis and necrosis. As shown in Fig. 6A,B (left side), compared to untreated groups, U373MG cells treated with HPM-M and HPM-PBS showed significant differences in apoptosis, whereas necrosis levels remained unchanged. HPM-DW groups showed the highest level of both apoptosis and necrosis in U373MG cells. However, U87MG cells treated with HPM-DW showed little increase in apoptosis, and no elevation in necrosis levels was observed (Fig. 6A,B, right side). In contrast to U373MG cells, HPM-M and HPM-PBS showed no significant increase in the levels of apoptosis and necrosis in U87MG cells. Cell death is tightly linked with membrane integrity, whereas apoptosis can be regulated by both extrinsic and intrinsic (mitochondrial) pathways. To further understand the membrane physiology in this context, we evaluated the changes in mitochondrial membrane potential using a Mitoflow Assay kit. Figure 6C indicates that U373MG cells showed changes in mitochondrial membrane potential when treated with HPM-DW compared to the control groups. Figure 6C indicates that U373MG cells undergo minor changes in membrane potential when treated with HPM-M and HPM-PBS compared to the control groups. However, no significant changes were observed in the HPM-M and HPM-PBS treatments in U373MG cells. In U87MG cells, the membrane potential in cells treated with all three HPM-irradiated solutions remained largely unaffected compared to the control groups. Several reports have indicated that NO and its derivatives can interact with mitochondria and affect mitochondrial respiration and its function^52–54^. This shows that induction of apoptosis pathways in HPM-DW groups could be due to changes in the mitochondrial membrane, which affects mitochondrial integrity and function, eventually leading to cell death.

### Changes in cell cycle dynamics with HPM-irradiated solutions

**Figure 7:**
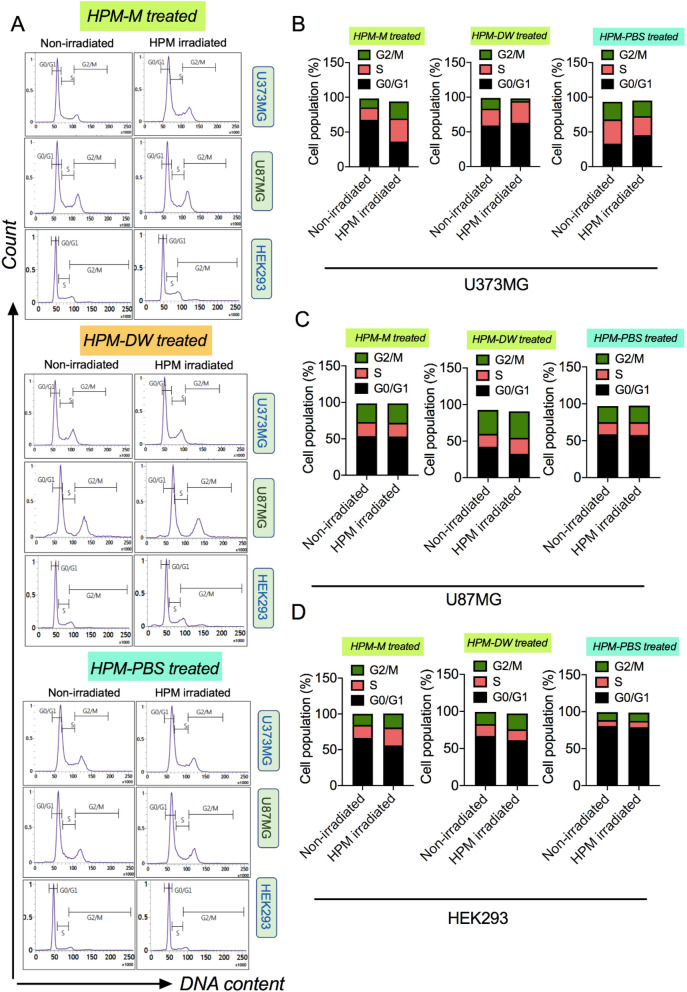
Cell cycle dynamics in cells after treatment with high power microwave-irradiated solutions. (**A**) Representative FACS histogram plots showing the cell populations of U373MG, U87MG, and HEK293 cells treated with high power microwave (HPM)-irradiated Media (upper side), HPM-irradiated DW (middle side), and HPM-irradiated PBS (lower side) in different cell cycle phases. (**B**–**D**) Percentage distribution of each cell population in the respective cell cycle stage of the cells shown in (**A**). The groups represented are cells treated with HPM-irradiated Media, DW, and PBS.

Another way to observe the cellular changes associated with cell death and apoptosis is to evaluate cell cycle arrest or changes in cell cycle phases. In this assay, cells were stained with propidium iodide solution (PI with ribonuclease A) overnight at 4 °C and subjected to flow cytometric analysis. Figure 7A shows the histogram depicting the cell cycle phases for cells treated with HPM-M (upper side), HPM-DW (middle side), and HPM-PBS (lower side) groups, and Fig. 7B,C,D show the corresponding percentage of cell population in the cell cycle phases of U373MG, U87MG, and HEK293 cells, respectively. In U373MG cells, HPM-DW-treated groups showed a decreased number of cells in the G2/M phase and an increased number of cells in the S and G1 phases compared to the control. In contrast, HPM-M-treated groups showed an increased number of cells in G2/M phase and reduced numbers in the G0/G1 phase compared to the control groups. HPM-PBS treated groups showed that the cell population increased marginally in G0/G1. U87MG cells showed a less profound effect on their cell cycle phases after treatment with HPM irradiated solutions. In U87MG cells , HPM-M and HPM-PBS treated groups did not show any alterations in cell cycle phases compared to the control groups, while the HPM-DW groups showed a marginal decrease in cell population in G0/G1 with increased cells in G2/M phase. Remarkably, in HEK293 cells, all three treatments showed very slight effects on cell cycle phases compared to the control groups. Overall, this study showed that HPM-DW treatment could induce arrest in the S phase and G1 phase of U373MG cells.

### HPM-irradiated solutions altered apoptotic genes at the transcriptional level

**Figure 8:**
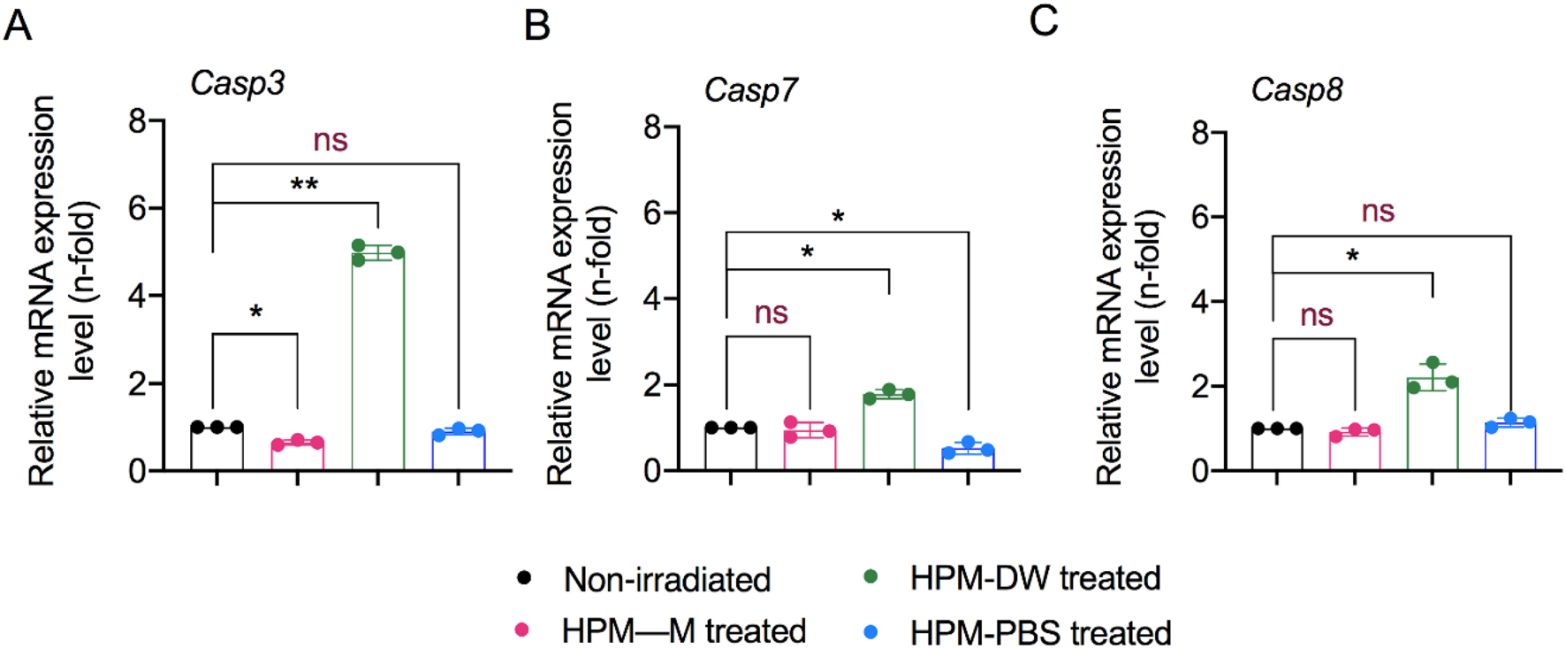
Effect of high power microwave-irradiated solutions on expression of cell death markers. Relative mRNA expression for apoptotic genes (**A**) Casp3, (**B**) Casp7, and (**C**) Casp8 in U373MG cells. The treatment groups are color-coded as shown in the figure. *p < 0.05, **p < 0.01, ***p < 0.001.

Cancer cells employ various strategies to evade cell death and related pathways, primarily by modulating pro-apoptotic genes and cell survival genes. It is thus important to assess changes at molecular levels related to cell death for biocompatibility and safety evaluations for treatment with any physical or chemical sources. Caspases are proteases that act as key regulators involved in the apoptotic process in cells. These genes are often dysregulated in cells undergoing stress at molecular levels, which may lead to cell death. We assessed whether HPM-irradiated solutions had any effect on the levels of caspases in cells. Therefore, we performed experiments to evaluate changes in the mRNA expression levels of key apoptotic pathway genes (*Casp3, Casp7, and Casp8*) in U373MG cells by real-time quantitative PCR analysis. The results shown in Fig. 8 indicate the expression levels of these apoptotic genes. All the comparisons are highlighted between cells treated with HPM-irradiated and control groups. *Casp3* expression was significantly increased in the HPM-DW treatment group as compared with the control (Fig. 8A). *Casp3* expression was slightly changed in both the HPM-PBS and HPM-M groups. In addition, *Casp7* expression remained unaffected or slightly changed in HPM-M—and HPM-PBS-treated cells. However, the HPM-DW treatment groups showed considerable elevation in the *Casp7* levels (Fig. 8B). We observed a significant increase in the expression of *Casp8* after treatment with HPM-DW, whereas there were no significant alterations in the HPM-M and HPM-PBS treated groups (Fig. 8C). Depending on the conditions and levels of ATP in cells, NO and peroxynitrite can modulate cell death in certain cell types. If ATP levels are low, cells may activate caspases, leading to apoptosis. However, if ATP levels are highly reduced, cells may inhibit apoptosis and activate necrosis. Activation of apoptosis by NO is also dependent on the levels of anti-oxidant biomolecules such as thiols and oxygen in the cells. Thiols scavenge peroxynitrite, whereas oxygen reduces the binding affinity between cytochrome oxidase and NO, preventing the breakdown of NO inside mitochondria. Here, we observed increased expression levels of caspases, indicating activation of apoptosis, particularly in U373MG cells treated with HPM-DW.

## Discussion

The ever-increasing use of MW often propels scientists to investigate its beneficial and harmful effects. Similar to light, MW can be transmitted, reflected, or absorbed by the target materials. Materials rich in water content such as processed foods, fluids, biological cells, or tissues tend to absorb MW energy promptly and convert it to heat. In the case of heating a liquid, the non-uniform heating behavior (non-convection method) of MW presents an interesting challenge for understanding and deciphering the interaction between biological solutions and MW^21^. Several studies indicate that the effect of MW on biological molecules present in solutions or inside cells and tissues cannot be induced similar to heating macroscopically^55–57^. The biological effects of MW can vary greatly and depend on two parameters: the physical properties of MW such as electric field strength, frequency, and duration of exposure^58,59^; and the properties of the material whether it is in solution or crystalline form^60^. The presence of unbound water molecules with the target material can affect the absorption of MW and changes in heating. In our recent study, we reported that direct exposure of cells in medium to HPM irradiation leads to non-thermal alterations in cells without impairing their growth^61^. Here, we observed no major changes in the temperature and pH of solutions after HPM irradiation, indicating that the resultant effect on cells could be non-thermal (Fig. 2). Interestingly, NO_x_ levels were elevated in HPM-DW and moderately increased in HPM-PBS, whereas they remained unchanged in HPM-M conditions. We propose a hypothesis wherein the generation of NO_x_ in liquids can be explained by the interaction between electric field induced by HPM and ambient air gases as well as water. Molecular nitrogen (N_2_), molecular oxygen (O_2_), and water vapor (H_2_O) are naturally present in the ambient atmosphere and interior of water. An electric field of approximately ~ 11 kV/cm corresponding to 30 kW of HPM interacts with the molecular nitrogen and oxygen present at the air and interior of water. These kinds of interaction convert them into their atomic nitrogen N and atomic oxygen O species. These may further combine to form NO_x_ that is incorporated into the liquid. The effect of HPM with electric field ~ 11 kV/cm used in this study stimulated the formation of NO_x_, as evidenced by its increase, as shown in Fig. 2J, which is similar to that appeared in nonthemal atmospheric pressure plasma jet^47^. Exogenous supplementation of these reactive NO_x_ groups can affect cellular NO homeostasis and may lead to significant alterations^62–64^. We also observed a slight increase in H_2_O_2_ levels in HPM-DW, whereas they remained unchanged in other groups. Such differences in NO_x_ and H_2_O_2_ levels can be explained by the composition and buffering capacity of PBS and media. Different results in HPM-PBS compared to the HPM-DW conditions could also be attributed to the endogenous antioxidant capacity of cells to neutralize the generated reactive species in U373MG cells.

The theoretical explanations for interactions between solutions in biological systems and MW seem to have limited possibilities owing to the dubious resonance excitation of biomolecules in solution^65^. Most of these explanations are well-summarized, giving clarity about such interactions with respect to proteins in solution^60,66,67^. MW energy exposure has been shown to affect cellular viability by altering protein physiology, gene expression, cellular growth pattern, and even cell death^68–70^. Our results show that HPM-Media and HPM-PBS had no major effect on cellular viability (Fig. 3). HPM-DW-treated cells underwent apoptosis as indicated by the apoptosis and necrosis assays in this study (Fig. 6). Further molecular investigations showed a major effect on the expression levels of apoptotic markers (Fig. 8). This indicates the possibility of caspase-dependent mechanisms differing from previous reports of caspase-independent pathways after MW exposure^71^. The mitochondrial membrane is an integral part of the cell and performs various physiological processes. MW radiation can damage the mitochondrial membrane in several ways. First, by increasing molecular rotation and thus increasing vibration, the collision frequency between molecules causes breakage of chemical bonds. This effect damages and compromises the structural integrity of the mitochondrial membrane^72^. Second, they act by increasing the intracellular reactive oxidative species and disrupting the regulation of antioxidant enzymes^73–75^. Third, phospholipases and proteases are activated by Ca^2+^ influx^76–78^. Mitochondria are the energy house of cells, and ATP production is their primary function. Mitochondrial dysfunction leads to reduced ATP levels^79^. In our study, we observed significant changes in the mitochondrial membrane potential in cells treated with HPM-DW, whereas no changes were observed in cells treated with HPM-M and HPM-PBS (Fig. 6). Mitochondrial dysfunction was further reinforced by significant changes in ATP levels in the HPM-DW groups (Fig. 4)^23^. Nitric oxide and its derivatives play a significant role in mitochondrial respiration. High levels or longer exposure to NO can cause reversible mitochondrial damage mainly by nitrosylation of key proteins, whereas peroxynitrite can cause irreversible oxidization and cross-linking of proteins, leading to mitochondrial membrane damage. Thus, NO-induced cytotoxicity is largely dependent on its derivatives. In our study, we believe NO may have played some role leading to observed changes. However, the exact underlying mechanism is not yet fully understood and needs to be examined further.

In addition, it cells treated with compounds such as ethanol/piperidine solutions in a MW irradiation environment can undergo cell cycle arrest and apoptosis^80^. A similar effect was also observed in NK cells when irradiated directly with MW^29^. Such an effect has not been evaluated for indirect exposure to HPM. Interestingly, we found that cells treated with HPM-irradiated solutions showed altered cell cycle phases marginally displaying increased cells in the S-phase (Fig. 7). In our study, we used HPM, which delivered high energy to cells, stimulating arrest in the S phase, and eventually leading to cell death in HPM-DW-treated U373MG cells. Previous studies have shown cell cycle arrest using low-power MW with high temperature^81^. Our study showed cell cycle alterations in U373MG cells, whereas U87MG cells remained unaffected. We believe these changes in the cell cycle could be due to increased NO in the HPM-DW condition. Previous studies that used NO donors have reported cell cycle arrest in the G0/G1 arrest due to modulation of cell cycle regulatory proteins^82,83^. Further, the differences in U373MG and U87MG cells may depend on the rate of DNA damage repair and the differential expression levels of key proteins involved in the cell cycle and cell death^84,85^. Moreover, U87MG cells have greater genomic stability when exposed to certain drugs or treatments as compared to U373MG cells^86^.

In conclusion, HPM was generated with a frequency of 3.5 GHz and power density 12 kW/cm^2^ at the sample position from an axial vircator, in which the electric field has been estimated to be ~ 11 kV/cm, which is found to be similar to ~ 10 kV/cm obtained from HFSS code, in both regions of air and DW inside the test tube. The effect of the HPM-irradiated DMEM cell culture medium, PBS, and DW was studied in cancer cells and normal cells by assessing the physiological changes. HPM irradiation with its electric field ~ 11 kV/cm led to changes in water, such as an increase in NO_x_ levels. Treatment of cancer (U373MG and U87MG) and normal (HEK293) cells with this irradiated water could induce changes in metabolic viability, mitochondrial membrane potential, and cell cycle, which eventually resulted in cell death, particularly in the U373MG cell population. On the contrary, the irradiated media and PBS did not induce any significant changes in cellular viability, energetics, and membrane physiology. Thus, HPM induced changes in the NOx levels of deionized water but not in media and PBS for activating the cell death cascade in U373MG cells. Understanding the reaction mechanisms during MW irradiation of solutions and water can thus facilitate the optimal use of MW in biological systems.

## Materials and methods

### HPM exposure device

Among HPM sources, the virtual cathode oscillator (vircator) is considered the best HPM source owing to its ability to generate MW with high power at a low voltage, the capability to operate at high frequencies, and ease of building and understanding. In this work, an axial vircator was constructed and operated for the generation of HPM. For this, a relativistic pulsed HPM generator named as “Chundoong,” was used; which uses a relativistic electron beam to generate high power HPM from several hundreds of megawatt to gigawatt levels^44,45^. In this device, a Marx generator is used to generate a high-voltage pulse, where 12 capacitors are connected in series. Each of the capacitors had a capacitance of up to 0.2 μF and were charged by a 20 kV DC supply for 1 min. They produce a pulse with high voltage when discharged in a series with a single trigger shot after charging. The characteristics of the emitted electron beam reached the maximum values (current of 88 kA, voltage of 600 kV, and pulse duration of 60 ns) when the characteristic impedance of the pulse-forming line, i.e., 6.8 Ω matched the diode chamber, which was evacuated with vacuum^44^.

### Cell culture

All human cell lines (HEK293, U87MG, and U373MG) were purchased from the Korean Cell Line Bank (Seoul, Korea). Cells were cultured in DMEM (Cat# LM001-05; Welgene, Korea) with 10% fetal bovine serum, 100 μg/mL of streptomycin, and 100 U/mL of penicillin, and were maintained in a humidified incubator with 5% CO_2_ at 37 °C. The cells were passaged every two–three days. The purpose of the use of these cell lines could be explained by the following explanation. (1) Brain is considered a very sensitive organ against MW radiatiom^26,87^. Glial cells are well distributed in the brain and regulate key responses during brain damage and also progression of brain cancer. U373MG and U87MG are human glioblastoma astrocytoma and primary glioblastoma cell line, respectively. On the other hand, HEK293 cells shares its cells of origin with neuronal cells and represents the normal cell counterpart.

### Nitric oxide (NO) assay

Total NO_2_^–^/NO_3_^–-^ was measured as an indicator of NO levels using the QuantiChrom Nitric Oxide Assay Kit (D2NO-100). All solutions (DMEM complete media, PBS, and deionized water) were placed in a glass tube and exposed to HPM radiation for 45 shots. The samples were collected and immediately processed following the manufacturer’s protocol. Eventually, the NO levels were assessed by measuring the absorbance at 540 nm using a Biotek microplate reader.

### Hydrogen peroxide (H_2_O_2_) assay

The QuantiChrom Peroxide Assay Kit (DIOX-250) was used to measure the levels of peroxides present in the solutions (DMEM complete media, PBS, and deionized water) after HPM irradiation. Similar to the NO experiment, the solutions were placed in a glass tube and exposed to HPM radiation for 45 shots. The samples were then collected and quickly processed following the kit protocol. The peroxide levels were analyzed by measuring the absorbance at 585 nm using a plate reader.

### Experimental setup

Each experiment consists of control, placebo and treatment groups. The untreated cells in media were considered as controls. The treatment groups consists of medium, PBS and DW exposed to HPM for 45 shots. Placebo groups consists of unexposed solutions (Medium, PBS and DW) which are kept in identical conditions until termination of 45 shots. Finally, the placebo group solutions and HPM-irradiated solutions were directly treated to cultured cells. During cell treatment, the volume was adjusted with complete medium to get final concentrations (5–100%). After 24 h of incubation, cells were harvested for viability, ATP and other assays.

### Metabolic viability

The metabolic viability of U373MG, U87MG, and HEK293 cells was determined using Alamar Blue (AB) dye (DAL1025; Thermo Fisher Scientific, Waltham, MA, USA). Briefly, cells were seeded at a density of 5 × 10^3^ cells in 100 µL per well of 96-well plates, followed by treatment with HPM-irradiated solutions on the next day. After incubation, an AB solution (10% v/v in medium) was added to each well and incubated for 2 h at 37 °C in a humidified incubator. Experiments were performed in triplicate or more with two groups: control and treatment (HPM-irradiated solutions) groups. Conversion of the AB dye was evaluated by measuring fluorescence emission using a BioTek plate reader with excitation/emission at 540/595 nm.

### Total ATP measurement

Cellular ATP as a measure of cellular health was measured using the Cell Titer-Glo Assay following the manufacturer’s instructions (Promega (cat no. G7572)). For this, 5 × 10^3^ cells per well (100 µL) were seeded in 96-well plates. Post 24 h of incubation, cells were supplemented with an equal volume of prewarmed reagent and incubated for 1 h at 37 °C. After incubation, luminescence was measured using a microplate reader.

### Flow cytometric analysis for cell death and cell cycle

To determine the levels of cell death in cells treated with HPM-irradiated solutions, cells were seeded at a density of 2 × 10^5^ cells/35 mm dish. The cells were treated and incubated for 24 h before being harvested and stained with propidium iodide (PI; Sigma Aldrich). The single cell suspensions thus obtained were assessed and analyzed by flow cytometry. For the cell cycle assay, 2 × 10^5^ cells/well were seeded and treated as previously described. After 24 h of incubation, cells were harvested, washed with cold PBS supplemented with 2% serum, fixed with ethanol (70%) for 1 h, and then pelleted down. The pellet was resuspended in PI staining solution (20 µg/mL PI and 50 µg/mL RNase A) and incubated overnight at 4 °C. The following day, cells were centrifuged, washed, and then resuspended in cold 1X PBS for sample acquisition with BD FACS Verse and analysis with FACS suite software.

### Intracellular nitric oxide (NO) assay

We measured intracellular nitic oxide levels using the Invitrogen Nitric Oxide Indicator (DAF-FM diacetate; Cat # D-23844). For intracellular NO measurement, 2 × 10^5^ cells/well were seeded and treated with HPM-irradiated solutions as previously described. Cells were incubated for 24 h followed by harvesting and washing with PBS, stained with DAF-FM-DCA following manufacturer’s protocol. After final incubation, sample acquisition was carried out with BD FACS Verse (excitation/emission : 495/515 nm) and analyzed with FACS suite software.

### Apoptosis and necrosis assays

To determine the mode of cell death pathways (apoptosis and necrosis), we evaluated cancer cells (U373MG and U87MG) using an Apoptosis Assay Kit (Promega; JA1011). Briefly, 5 × 10^3^ cells per well (100 µL) were seeded in 96-well plates for treatment and incubated for 24 h. After incubation, cells were supplemented with an equal volume of prewarmed 2X detection reagent and incubated for 1 h at 37 °C. After incubation, luminescence and fluorescence (excitation/emission of 485/525 nm) were measured using a microplate reader to indicate apoptosis and necrosis, respectively.

### Quantitative real time qPCR

For quantification of mRNA expression, total RNA was extracted from control and treatment groups using RNAiso Plus (Takara) following the manufacturer’ s protocol. Total RNA (2 µg) was used to synthesize the template DNA using Reverse Transcription Supermix (Enzynomics) and the provided protocol. The reverse transcription supermix consisted of MMLV RT buffer (Tris–HCl [pH 8.3]) (500 mM), MgCl_2_ (30 mM), DTT (100 mM), KCl (750 mM)), dNTPs, MMLV reverse transcriptase, and RNase inhibitor. iQ SYBR Green Supermix (Bio-Rad) was then used to prepare the reactions followed by running on an iCycler IQ real-time detection system (Bio-Rad, USA). The primers used in this study were designed and procured from DNA Macrogen, Korea. Primer sequences are provided in Table 1.

**Table 1:** Primer sequences used in this study.

Gene Name	Sequence (5′–3′)	Size (bp)
ACTIN-forward	GGC ATC CTC ACC CTG AAG TA	82
ACTIN-reverse	AGG TGT GGT GCC AGA TTT TC	
Casp3-forward	ATG TCG ATG CAG CAA ACC TC	173
Casp3-reverse	TCC TTC TTC ACC ATG GCT CA	
Casp7-forward	AGA AGC TGT TAC CAC ACC CA	185
Casp7-reverse	ACT CCA TCT CAG TCA GTG GC	
Casp8-forward	CCC AAA TCA ACA AGA GCC TGC	219
Casp8-reverse	TCA GAC AGT ATC CCC GAG GTT	

### Statistics

The results are expressed as the mean ± SD of triplicate experiments from three independent experiments. Student's *t*-tests were used to analyze significant differences between groups, whereas one-way ANOVA was used for comparison between multiple groups. Significance was considered as *p < 0.05, **p < 0.01, and ***p < 0.001. Non-significant groups were denoted as ns.

## Supplementary Information

Supplementary Information
