# Supplementary material for: Pulsed 3.5 GHz high power microwaves irradiation on physiological solution and their biological evaluation on human cell lines
Source: Sci Rep. 2021 Apr 19;11:8475. doi: 10.1038/s41598-021-88078-x (PMC8055702; doi:10.1038/s41598-021-88078-x)
Supplement: Supplementary file 1 — Supplementary Information [file 41598_2021_88078_MOESM1_ESM.pdf]

Supplementary Information

Pulsed 3.5 GHz high power microwaves irradiation on physiological solution and their biological evaluation on human cell lines

Pradeep Bhartiya[1], Sohail Mumtaz[1], Neha Kaushik[2], Pradeep Lamichhane[1], Linh Nhat Nguyen[1], Jung Hyun Jang[1], Nagendra Kumar Kaushik[1]*, Eun Ha Choi[1]*

[1] *Plasma Bioscience Research Center/Applied Plasma Medicine Center, Department of Plasma Bio Display, Department of Electrical and Biological Physics, Kwangwoon University, Seoul, 01897, Korea*

[2] *College of Engineering, Department of Biotechnology, University of Suwon, Hwaseong, 18323, Korea*

Corresponding author: ehchoi@kw.ac.kr & kaushik.nagendra@kw.ac.kr

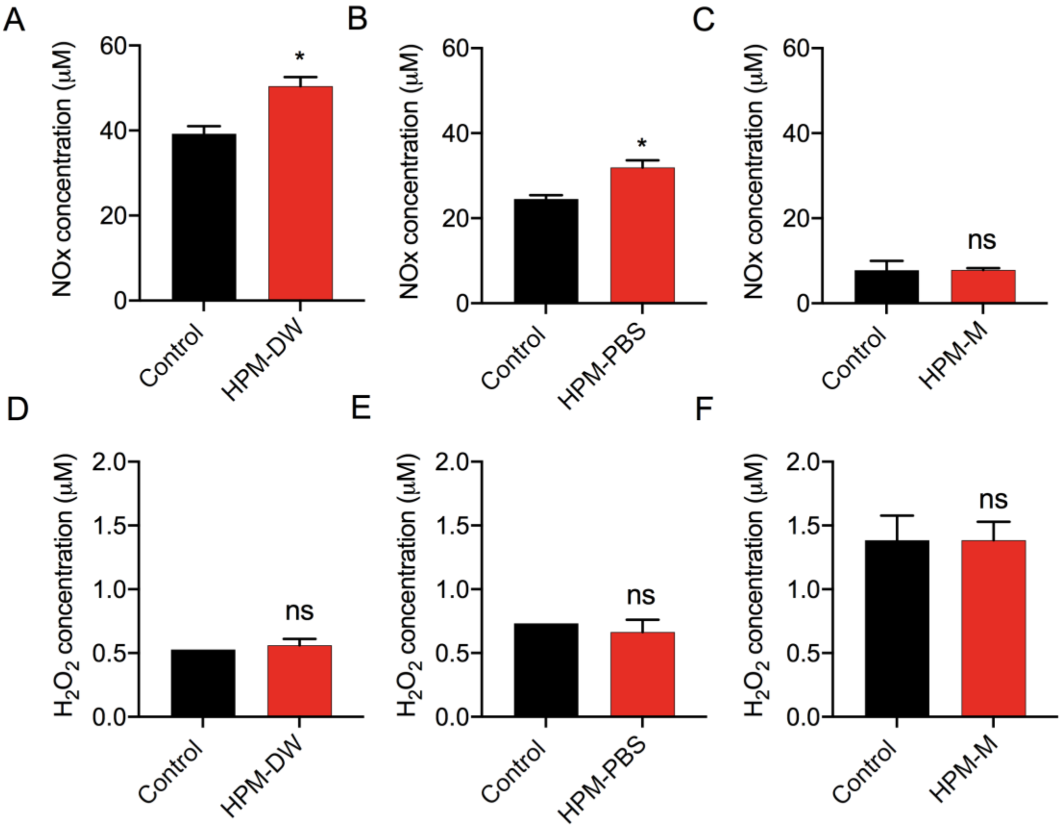

Fig S1. Evaluation of reactive species after HPM-irradiation of solutions. Representative graphs showing levels of NO_x in A) HPM-DW B) HPM-PBS and C) HPM-Medium. Respresntaive graphs showing levels of H_2O_2 in D) HPM-DW E) HPM-PBS and F) HPM-Medium. Non-significance is signified as ns. *$p < 0.05$.

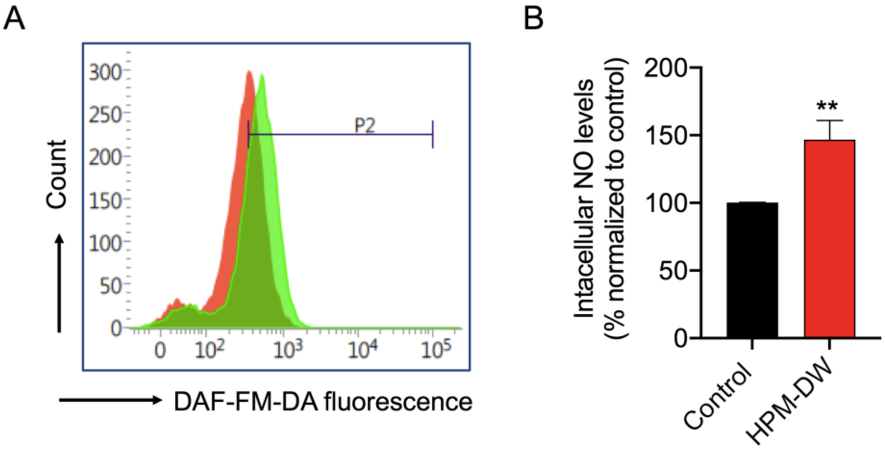

Fig S2. Effect of HPM-irradiated solutions on intracellular NO levels. Representative histogram (A) and corresponding bar graph (B) showing DAF-FM-DA fluorescence as an indicator of intracellular NO in U373MG cells stained with DAF-FM Diacetate. **$p < 0.01$
